# 

**Published:** 1857-06

**Authors:** 


ARTICLE I.—An Address, Introductory to the Third Course of Lectures in the Atlanta Medical College. By J. BORING, M. D., Professor of Obstretrics, &c., Atlanta, Ga, ..... 577

ARTICLE II.—Foreign Correspondence. By W. F. WESTMORELAND, M. D., Professor of the Principles and Practice of Surgery in the Atlanta Med. College, ..... 591

ARTICLE III.—Aneurism of the Brachial Artery cured by injection of per chloride of Iron. By M. LAGRANGE, Surgeon-in-Chief of the Civil and Military Hospital, at St. Mibriel, (Mense.) Translated by J. J. WEST, M. D., Savannah, Ga., ..... 600

### SELECTIONS.

A Monograph on Ovarian Tumors, ..... 611

Translation from Foreign Journals, ..... 623

A case of Excision of the Hip-joint for Morbus Coxarius, ..... 625

### EDITORIAL AND MISCELLANEOUS.

A trip to Tennessee, ..... 631

Atlanta Medical College, ..... 632

Savannah Medical College, ..... 633

American Medical Association, ..... 634

University of Nashville, ..... 535

Books, &c., received, ..... 635

Address of K. R. WINSTON, M. D., ..... 636

Hydrocele cured by friction of the Tunica Vaginalis, ..... 637

Glycerine in Phthisis, ..... 638

Singular case of Triplets ..... 638

Ill effects of Quinine, ..... 638

William Lawrence, ..... 639

Meteorological Observations for April and May, 1857, at Atlanta, Ga., ..... 639 640

Dr Kane, ..... 640

